# Expert opinion: Radiotherapy in cutaneous squamous cell carcinoma: Recommendations from the DEGRO dermatooncology working group

**DOI:** 10.1007/s00066-026-02559-4

**Published:** 2026-06-24

**Authors:** Kathrin Hering, Panagiotis Balermpas, Alexander Rühle, Maike Trommer, Khaled Elsayad, Sebastian Adeberg, Dirk Vordermark

**Affiliations:** 1 Department of Radiotherapy and Radiation Oncology, University Hospital Leipzig, Leipzig, Germany; 2 Comprehensive Cancer Center Central Germany, Partner Site Leipzig, Leipzig, Germany; 3 Department of Radiation Oncology, University Hospital Zurich, University of Zurich, Zurich, Switzerland; 4 Department of Radiation Oncology, Medical Center, Faculty of Medicine, University of Freiburg, Freiburg, Germany; 5 Department of Radiation Oncology, Faculty of Medicine, University Hospital Bonn, Bonn, Germany; 6 Department I of Internal Medicine, Center of Integrated Oncology Aachen Bonn Cologne Düsseldorf, University Hospital of Cologne, Cologne, Germany; 7 Department of Radiotherapy and Radiation Oncology, Philipps-Universität Marburg, Marburg, Germany; 8 Marburg Ion-Beam Therapy Center (MIT), Department of Radiotherapy and Radiation Oncology, Marburg University Hospital, Marburg, Germany; 9 University Cancer Center (UCT) Frankfurt-Marburg, Marburg, Germany; 10 Department of Radiation Oncology, University Hospital Halle (Saale), Halle (Saale), Germany

## Introduction

Keratinocyte carcinoma (KC), formerly known as non-melanoma skin cancer (NMSC), includes basal cell carcinoma (BCC), cutaneous squamous cell carcinoma (cSCC), and their precursors such as actinic keratosis (AK) and Bowen’s disease, an in situ form of cSCC [1]. cSCC is the second most common form of skin cancer after BCC, accounting for approximately 20% of KC [2, 3]. Determining its precise incidence is challenging; however, cSCC represents a growing public health concern, with recent data showing a sustained increase in incidence in Germany [4]. Known risk factors are fair or light skin, male sex, cumulative ultraviolet radiation exposure, history of severe sunburns, older age, systemic immunosuppression (including organ transplantation and hematologic malignancies such as chronic lymphocytic leukemia), smoking, and genetic predisposition such as xeroderma pigmentosum [5–7].

Most patients with cSCC have an excellent prognosis and are cured by surgery. Therefore, surgery remains the standard of care for cSCC. However, radiotherapy (RT) represents an effective alternative treatment option, particularly in cases where surgery is contraindicated; technically challenging due to anatomical constraints such as in the periorbital region, nose or ears; or is declined by the patient. In patients with hereditary syndromes conferring increased radiosensitivity, RT is conditionally discouraged. The very low risk of radiation-induced in-field malignancy in the future among patients younger than 60 years should also be considered [8]. However, RT provides excellent local control rates and is associated with favorable cosmetic and functional outcomes [9]. Quality of life can be preserved or improved in subdomains such as emotions, functioning, social relations, and personal appearance in patients with early-stage disease after RT [10, 11]. For the treatment of cSCC, RT includes electron beam RT, superficial x‑ray therapy (SXRT), orthovoltage RT, brachytherapy, and megavoltage photon (external beam) RT. The choice of fractionation schedule and RT modality, defined by specific radiophysical characteristics affecting tissue penetration and dose distribution, is guided by clinical parameters including tumor thickness, anatomical limitations, and patient-specific factors. Therapeutic recommendations, RT delivery techniques, and fractionation considerations for primary and postoperative RT are provided by national and international guidelines for cSCC [8, 12–15]. Immunotherapy, particularly PD‑1 inhibitors like cemiplimab, is emerging as a promising approach for neoadjuvant and adjuvant high-risk cSCC, but its optimal use is still being defined [16–18]. This recommendation aims to provide an update on the current use of RT in the treatment of invasive cSCC.

## Radiobiological effects of radiotherapy

The mechanism of RT action involves induction of indirect and direct cellular DNA damage, primarily through double-strand breaks (DSBs), which can lead to cellular apoptosis if the damage is irreparable. Indirect effects are mediated by the formation of reactive oxygen species (ROS) from the radiolysis of water, which further damages DNA and other cellular components. In cSCC, radiation induces apoptosis, mitotic catastrophe, and senescence in tumor cells, while also stimulating immune responses through the release of tumor antigens [19, 20]. Normal skin structures, including basal keratinocytes, hair follicles, and sebaceous glands, are also affected, potentially leading to acute side effects such as erythema and desquamation and late effects like fibrosis or telangiectasia. Fractionated RT exploits these principles by allowing normal tissues to repair sublethal damage between doses while maximizing tumor control.

## Current treatment modalities (literature review)

### Primary definitive radiotherapy

**Fig. 1:**
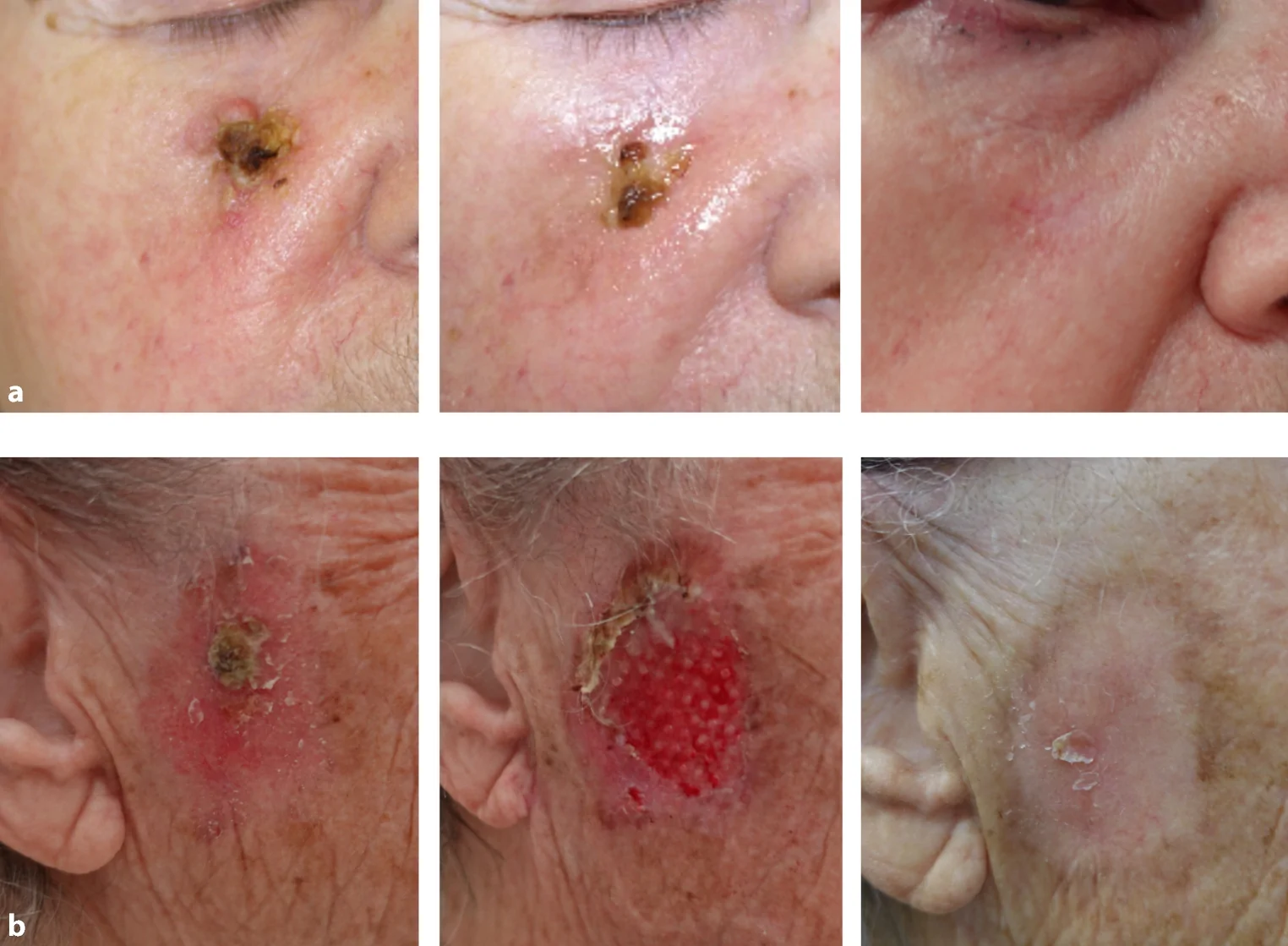
Curative radiotherapy for cutaneous squamous cell carcinoma (cSCC) as a single modality. **a** An 82-year-old female patient with cSCC on the right infraorbital area before, at the end of treatment, and 2 years after treatment following brachytherapy delivered as 8 × 4 Gy twice a week using an individualized moulage. **b** An 84-year-old female patient with cSCC on the right preauricular area before, at the end of treatment, and 2 years after treatment following brachytherapy delivered as 8 × 4 Gy twice a week using a Freiburg flap

Definitive RT is recommended as a curative treatment modality in patients with cSCC who cannot undergo or decline surgical resection, or in anatomic locations where surgery can compromise function or cosmesis. The increasing significance of shared decision-making is especially important for many cSCC patients, particularly in situations where surgery might lead to cosmetic or functional impairments, such as those involving the nose, ear, eyelid, or lip (Fig. 1). In these cases, radiotherapy can be considered to prevent facial disfigurement, loss of function, or prolonged wound healing. In patients with genetic diseases predisposing to heightened radiosensitivity, e.g., basal cell nevus syndrome (Gorlin syndrome), definitive RT is conditionally not recommended. In patients with locally advanced cSCC, the addition of concurrent drug therapies to definitive RT is conditionally recommended [8], although randomized phase III trials are lacking. Neoadjuvant cemiplimab may be considered as a treatment option for patients with inoperable or borderline-resectable cSCC, followed by surgery or—for non-surgical candidates—definitive RT. However, data on the sequencing of neoadjuvant cemiplimab followed by RT are scarce, and prospective trials are needed to determine the optimal treatment combination and sequence [18, 29]. Contrary to the common misconception that tumors with extensive bone involvement have poorer local control with RT, evidence from multiple studies demonstrates benefits [21–24]. Inclusion of the course of a cranial nerve should be considered for extensive perineural invasion (PNI) or involvement of a named cranial nerve. If there is clinical or radiological perineural tumor spread (PNTS), comprehensive coverage of the involved nerve is recommended, even though the risk of chronic high-grade adverse events is increased.

A variety of RT techniques and dose and fractionation schemes are reported in the current guidelines (Table 1; [8, 12–15, 25, 26]). Treatment planning must optimize tumor control while preserving cosmetic appearance and functional integrity. Electron beam RT, SXRT, orthovoltage RT, and brachytherapy are beneficial for superficial lesions and tumors located in sensitive anatomical areas such as the nose and ear, whereas deeper tumors or those with perineural invasion require photon external beam RT. For fractionation, especially in the older patient population, there is a trend toward hypofractionated treatment regimens.

**Table 1:** Primary definitive radiotherapy [8, 12–14]

*Conventional fractionation*	BED_10_ 70–93 Gy	60–64 Gy for small tumors (< 2 cm)
		66–70 Gy
*Hypofractionation*	BED_10_ 56–88 Gy	2.5 Gy–3 Gy × 20–22 fractions
		4.4 Gy × 10 fractions; 4 fractions per week
		6–8 Gy × 5 fractions; 2–3 fractions per week
*Brachytherapy*	BED_10_ ≥ 60 Gy	*Custom surface mold applicators (prescription depth ≤* *5* *mm)*
		3 Gy × 16 fractions, daily
		5 Gy × 8 fractions, 2 fractions per week
		*Interstitial brachytherapy (prescription depth <* *5* *mm)*
		4.4 Gy × 10 fractions, twice daily
		*Shielded single channel surface applicators (Leipzig or Valencia; prescription depth ≤* *3* *mm)*
		3 Gy × 16 fractions, daily

*BED*_*10*_ Biologically effective dose (α/β = 10)

### Postoperative radiotherapy

One key decision after surgery of cSCC is whether to recommend postoperative radiation therapy (PORT) (Table 2). Various factors may contribute to an aggressive course of cSCC, including pathological features such as close or positive margins, moderate to poor differentiation, and extensive perineural or lymphovascular invasion, as well as clinical features like immunosuppression and recurrent disease. In patients with a high risk of recurrence, current guidelines recommend multidisciplinary consultation and consideration of PORT. The addition of concurrent chemotherapy to PORT is not recommended [27]. Postoperative radiotherapy is recommended for close or positive margins, if re-resection is not possible, for extensive lymph node involvement (> 1 affected lymph node, lymph node metastasis > 3 cm, and extracapsular spread), for extensive perineural invasion, and for intraparotid lymph nodes [13]. ASTRO guidelines further recommend PORT for AJCC 8th edition T3 and T4 tumors, for desmoplastic or infiltrative tumors and in the setting of chronic immunosuppression [8].

Postoperative radiotherapy offers benefit in cases of microscopically positive margins (R1) by reducing the risk of local recurrence, though the magnitude of this benefit remains debated [28].

The addition of concurrent chemotherapy to PORT is not recommended [13, 27]. Nevertheless, recent randomized, phase III data demonstrate significantly improved outcomes for the combination of RT and the PD-1-antibody cemiplimab in the postoperative setting for high-risk situations (extracapsular extension with largest node ≥ 20 mm in diameter or at least three involved nodes) or non-nodal features (in-transit metastases, T4 lesion [with bone invasion], perineural invasion, or locally recurrent tumor with ≥ 1 additional risk features). Importantly, in this trial, the addition of systemic therapy administered after PORT did not replace radiotherapy [16]. Currently, there are no randomized phase III trials that directly compare neoadjuvant and adjuvant immunotherapy for cutaneous squamous cell carcinoma (cSCC). The phase II EMPOWER-CSCC‑1 study did not find a significant difference between the neoadjuvant and adjuvant immunotherapy cohorts. The sequence of immunotherapy and radiotherapy should be discussed in a multidisciplinary tumor board, considering the specific clinical scenario and the operability status of the patient [29].

**Table 2:** Postoperative radiotherapy [8, 12–14]

*Conventional fractionation*	BED_10_ 60–79 Gy	60–66 Gy
*Hypofractionation*	BED_10_ 48–70 Gy	2.5 Gy × 20 fractions
		3 Gy × 15–20 fractions
		6 Gy × 5 fractions (non-consecutive days)

*BED*_*10*_ Biologically effective dose (α/β = 10)

### Radiotherapy for treating regional nodes and regional disease management

For patients with disease that has metastasized to clinically apparent regional lymph nodes, therapeutic lymphadenectomy followed by adjuvant RT is recommended, with the exception of patients who have a single small (<3 cm) cervical lymph node harboring carcinoma without extracapsular extension. Definitive RT to regional lymph nodes is only recommended for patients who are medically inoperable or surgically unresectable. No data or general recommendations exist for elective nodal irradiation. For patients at a high risk of regional nodal metastasis (e.g., thickness >6 mm, poor differentiation, immunosuppression), elective lymph node basin RT is conditionally recommended only for those undergoing RT to the primary site with overlap of the adjacent nodal basin ([8, 12–15]; Table 3).

**Table 3:** Radiotherapy to the regional nodal basin [8, 12–14]

*Conventional fractionation*	50–60 Gy (lymph node and dissected nodal basins)
	50–66 Gy (perineural tumor spread)
	66–70 Gy (gross residual lymph nodes)

## Recommendation

Radiotherapy is an effective curative or adjuvant modality for cSCC, particularly in patients who are medically inoperable or where surgery would cause unacceptable cosmetic or functional impairment. Adjuvant RT is recommended for cases with high-risk features of local recurrence, including positive or close margins, locally recurrent tumor, perineural invasion of a gross nerve, lymphovascular invasion, or nodal involvement. The treatment technique and dose selection depend on tumor depth and the anatomical location. Superficial orthovoltage therapy is appropriate for lesions ≤5 mm thick, while electron beam therapy is preferred for deeper lesions. For deeply infiltrative tumors or postoperative cases, photon-based radiation provides conformal coverage with optimal organ sparing. High-dose-rate (HDR) brachytherapy is suitable for small, well-defined lesions, particularly on the lip, nose, or eyelid. Typical definitive RT schedules include 66–70 Gy in 2‑Gy fractions or hypofractionated regimens particularly for elderly or frail patients. Postoperative RT is commonly prescribed with 60–66 Gy to the tumor bed and 50–54 Gy to nodal areas, importantly in combination with anti-PD1 treatment (and not as an alternative) for high-risk situations. Proper patient selection and technique ensure excellent tumor control and cosmetic outcomes.

## Conclusion

Radiotherapy represents an excellent treatment option for cSCC. Adherence to guideline-based recommendations regarding indications for definitive and postoperative RT, including appropriate selection of modality and fractionation, facilitates the tailoring of treatment to complex anatomical constraints and individual patient factors, thereby optimizing local tumor control. The future of RT in cSCC is advancing rapidly, driven by innovations in precision RT, integration with systemic and immunotherapeutic approaches, and a growing emphasis on personalized oncology care.

## Data Availability

All data supporting the findings of this work are included in the article.
